# Burrow characteristics of the co-existing sibling species *Mus booduga *and *Mus terricolor *and the genetic basis of adaptation to hypoxic/hypercapnic stress

**DOI:** 10.1186/1472-6785-9-6

**Published:** 2009-04-09

**Authors:** Sunita Singh, Nge Cheong, Gopeshwar Narayan, T Sharma

**Affiliations:** 1 Department of Zoology, Mahila Mahavidyalaya, Banaras Hindu University, Varanasi – 221005, India; 2 Bioprocessing Technology Centre, Clinical Research Centre, Department of Pediatrics, Faculty of Medicine, National University of Singapore – 119 260, Singapore; 3 Department of Molecular and Human Genetics, Banaras Hindu University, Varanasi – 221005, India; 4 Cytogenetics Laboratory, Department of Zoology, Banaras Hindu University, Varanasi – 221 005, India

## Abstract

**Background:**

The co-existing, sibling species *Mus booduga *and *Mus terricolor *show a difference in site-preference for burrows. The former build them in flat portion of the fields while the latter make burrows in earthen mounds raised for holding water in cultivated fields. In northern India which experiences great variation in climatic condition between summer and winter, *M. booduga *burrows have an average depth of 41 cm, as against 30 cm in southern India with less climatic fluctuation.

*M. terricolor *burrows are about 20 cm deep everywhere. The three chromosomal species *M. terricolor *I, II and III have identical burrows, including location of the nest which is situated at the highest position. In contrast, in *M*. *booduga *burrows, the nest is at the lowest position.

**Results:**

The nest chamber of *M. booduga *is located at greater depth than the nest chamber of *M. terricolor*. Also, in the burrows of *M. booduga *the exchange of air takes place only from one side (top surface) in contrast to the burrows of *M. terricolor *where air exchange is through three sides. Hence, *M. booduga *lives in relatively more hypoxic and hypercapnic conditions than *M. terricolor*.

We observed the fixation of alternative alleles in *M*. *booduga *and *M*. *terricolor *at Superoxide dismutase-1 (Sod-1), Transferrin (Trf) and Hemoglobin beta chain (Hbb) loci. All the three are directly or indirectly dependent on oxygen concentration for function. In addition to these, there are differences in burrow patterns and site-preference for burrows suggesting difference in probable adaptive strategy in these co-existing sibling species.

**Conclusion:**

The burrow structure and depth of nest of the chromosomal species *M. terricolor *I, II and III are same everywhere probably due to the recency of their evolutionary divergence. Moreover, there is lack of competition for the well-adapted 'microhabitats' since they are non-overlapping in distribution. However, the co-existing sibling species *M*. *booduga *and *M*. *terricolor *exhibit mutual "exclusion" of the 'microhabitats' for burrow construction. Thus, location, structure and depth of the burrows might have been the contributory factors for selection of alternative alleles at three loci Sod-1, Trf and Hbb, which reflect difference in probable adaptive strategy in *M. booduga *and *M. terricolor*.

## Background

*Mus booduga *(Gray 1837) and *Mus terricolor *Blyth, 1851 are the indigenous pygmy field mice of India and are highly interesting for evolutionary studies. They are morphologically alike and were considered conspecific until Matthey and Petter [1] discovered that they were two sibling species, primarily based on their divergent karyotypes. These co-existing, sibling species are closely allied to the house mouse *Mus musculus *belonging to a sister lineage. They are found in abundance in cultivated fields being a major pest infesting mainly rice and wheat fields. These field mice have 2n = 40 chromosomes as in other *Mus *species. *M. booduga *has the same karyotype throughout its distribution with all acrocentric chromosomes in the complement, identical to that of *M. musculus*. The karyotype of *M. terricolor*, on the other hand, is distinct due to the presence of large submetacentric X and large acrocentric Y chromosomes. In addition, different apparently non-overlapping populations of *M. terricolor *possess divergent karyotypes showing variation in the number of autosomal heterochromatic large short arms established in homozygous condition [2,3]. A multidimentional investigation carried out over the years in our laboratory at Varanasi revealed recency of evolutionary differentiation of the three chromosomal species of *M. terricolor *which however have developed both pre-mating and almost complete post-zygotic isolations [3-7].

We have earlier analysed 20 enzymatic and nonenzymatic loci in *Mus booduga-terricolor *complex as well as *Mus musculus tytleri *[6] for genetic variations. Among 20 loci only three loci (hemoglobin beta-chain, transferrin and superoxide dismutase) showed adaptive correlation. We have observed direct correlation of the difference in preference for sites for burrows in the fields and difference in depth of burrows and nest chamber position in the burrows of *M*. *booduga *and *M. terricolor *to the fixation of alternative alleles at three loci hemoglobin beta-chain (Hbb), transferrin (Trf) and superoxide dismutase (Sod-1) (whose functions are directly or indirectly dependent on oxygen concentration). The fixation of alternative alleles at these three loci, and the differences in their burrow patterns and location probably reflect adaptive strategy difference between the co-existing, sibling species.

## Results

The season of breeding of both *M*. *terricolor *and *M*. *booduga *coincided with the harvesting of major crops, namely, rice and wheat. In southern India, where the difference in climatic conditions in summer is less-marked compared to other seasons, and availability of the crops is fairly continuous, breeding was observed almost throughout the year. In the north however, these mice were hardly traceable in the parched fields during hot summer months, but they were in abundance when rice and wheat were harvested in October/November and March/April. These pygmy field mice remain in burrows during day time and come to surface only at nights in search of food and mates.

### Burrow study

A characteristic feature of live burrows was the presence of small mounds of minute damp soil pellets at the entrance. Larger mounds were seen at the entrance of breeding or family burrows with pups. Pups were found in globular nests padded with dried grass and straw. A study of 76, 14 and 73 litter of *M*. *terricolor *I, II and III, respectively showed that the litter size varied from 2 to 19 with a mean of 8. Seven litters of *M. booduga *were examined and were found to be similar in size as in *M*. *terricolor*.

**Figure 1:**
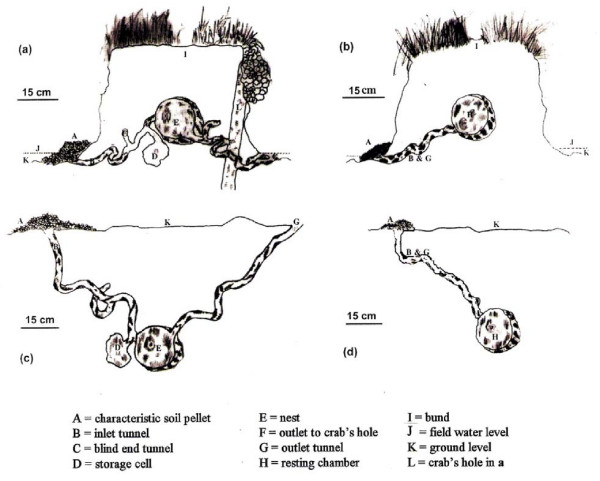
**Diagrammatic representation of family (a) and male (b) burrows of *M. terricolor*; family (c) and male (d) burrows of *M. booduga***.

The presence of mice in burrows could be ascertained by the degree of freshness or wetness of pellets at the entrance. The entrance and particularly the exit of the burrows were camouflaged making them fairly inconspicuous. It was striking that *M*. *terricolor *constructed their burrows in the raised earthen mounds or bunds built for holding water in cultivated fields. The *M*. *booduga *burrows, on the other hand, were located in flat portions of the fields. The difference for preference of site for making burrows was found in northen as well as southern India. A total of 98 burrows of *M*. *booduga *(24 from Varanasi, 20 from Erode and Mysore and 54 from Chennai) and 194 burrows of the *M. terricolor *complex (83 of *M. terricolor *I from Varanasi, 13 of *M*. *terricolor *II from Mysore and 98 of *M*. *terricolor *III from Chennai) were excavated and studied. The burrows of *M*. *booduga *and *M. terricolor *had similarities in diameter (2.5 cm) of inlet and outlet tunnels, diameter (13–14 cm) of nest chamber, number of storage cell (usually one but not exceeding two in a burrow) and in having a single entrance (table 1, fig. 1).

**Table 1:** Average burrow dimensions of Indian field pigmy mice.

Taxa (no. of burrows)	Length of inlet tunnel (Range) (± SD)	Length of outlet tunnel (Range) (± SD)	Depth of Nest chamber (Range) (± SD)	Diameter of nest	Diameter of tunnel (inlet and outlet)
*M. booduga *(V) (24)	66 cm (40 – 80 cm) (± 10.90)	77 cm (30–90 cm) (± 15.30)	41 cm (38 – 45 cm) (± 1.96)	13 – 14 cm	2.5 cm
*M. booduga *(M) (74)	46 cm (20 – 50 cm) (± 6.90)	43 cm (10 – 50 cm) (± 9.92)	30 cm (28 – 32 cm) (± 1.41)	13 – 14 cm	2.5 cm
*M. terricolor *I (83)	45 cm (30 – 76 cm) (± 10.70)	50 cm (40–70 cm) (± 7.92)	20 cm (18 – 22 cm) (± 1.44)	13 – 14 cm	2.5 cm
*M. terricolor *II (13)	45 cm (30 – 76 cm) (± 14.00)	50 cm (40–70 cm) (± 8.98)	20 cm (18 – 22 cm) (± 1.53)	13 – 14 cm	2.5 cm
*M. terricolor *III (98)	45 cm (30 – 76 cm) (± 10.90)	50 cm (40–70 cm) (± 7.99)	20 cm (18 – 22 cm) (± 1.40)	13 – 14 cm	2.5 cm
ANOVA (F-value)	19.84	50.86	1187	-	-

The burrows of *M*. *terricolor *I, II and III showed more or less similar measurements and other features in different soil-types from northern and southern India. It was interesting that *M. terricolor *seem to prefer moist soils, and often one would come across tunnel of crabs by the side of *M. terricolor *burrows (fig. 1a). The average lengths of the inlet and outlet tunnels were almost equal being 45 cm (range: 30–76 cm) and 50 cm (range: 40–70 cm) respectively. The nest chamber was at an average 20 cm (range: 18–22 cm) from the top surface which was markedly less deeper compared to the nest chamber in *M*. *booduga *burrows which was at an average depth of 41 cm (range: 38–45) in the alluvial and deltaic soils in the north and at 30 cm (range: 28–32 cm) in the red and coastal alluvial soils in the south. The burrows in northen India therefore had longer and deeper tunnels; the inlet and outlet tunnels being 66 cm (range: 40–80 cm) and 77 cm (range: 30–90 cm) as compared to shallower 46 cm (range: 20–50 cm) and 43 cm (range: 10–50 cm) ones in southern India (table 1). Analysis of variance (ANOVA) shows high F-values indicating significant difference among taxa with respect to lengths of inlet and outlet tunnels and the depth of the nest chamber (table 1). Thus, the difference in location of the burrows and in position of the nest chamber, (which is at a level lower than the inlet and outlet tunnels in *M*. *booduga *whereas in *M*. *terricolor *it is situated above the levels of the inlet and outlet tunnels), would affect concentration of O_2 _and CO_2 _in the nest chambers of their burrows.

Besides the family burrows with females and pups, much simple burrows were also found which were occupied only by adult male. Excavation of 17 such burrows of both *M*. *terricolor *(fig. 1b) and *M. booduga *(fig. 1d) showed that these had no separate outlet tunnels, storage cell or blunt-end tunnel. The inlet tunnel of each species ended in a chamber resembling the nest chamber, but without bedding. The inlet tunnel was running downward from the entrance in case of *M*. *booduga *burrows whereas it was running upward in *M*. *terricolor *burrows. The sites for the male burrows were as for the family/breeding burrows.

### Electrophoretic analysis

**Figure 2:**
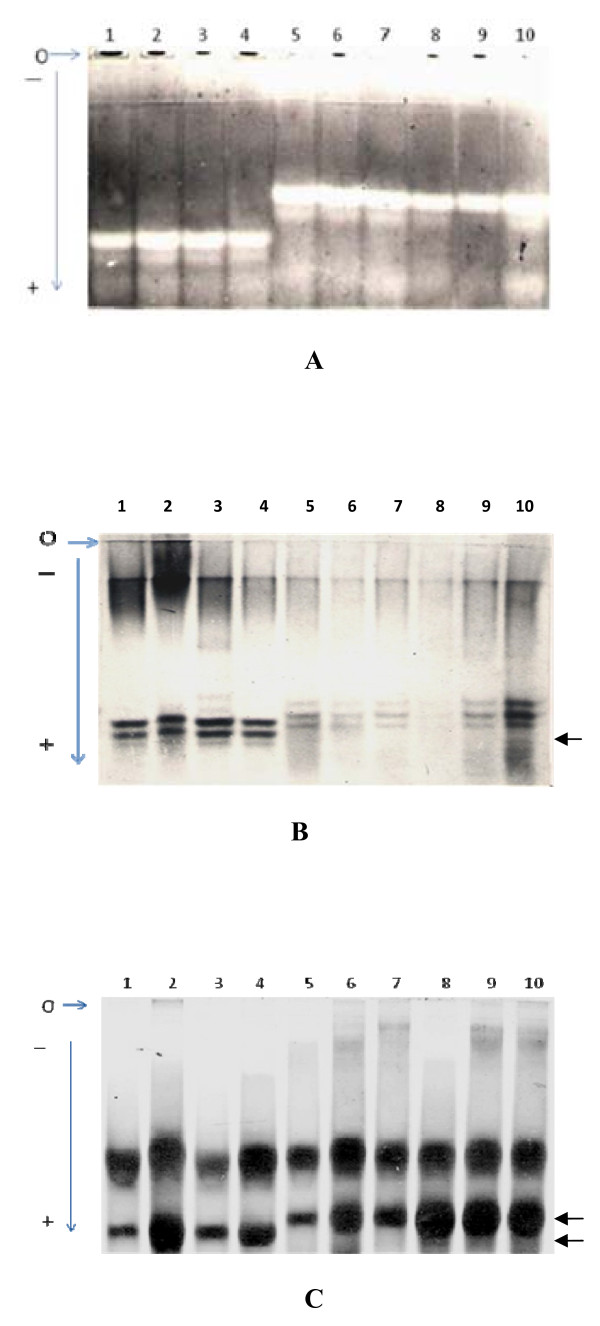
**A. Zymogram of Superoxide dismutase (Sod-1)**. Lanes 1, 2 *M. booduga *(M); Lanes 3, 4 *M. booduga *(V); Lanes 5, 6 *M. terricolor *I; Lanes 7, 8 *M. terricolor *II; Lanes 9, 10 *M*. *terricolor *III. Lanes 1 to 4 show bands of faster mobility (Sod-1^b^) in *M. booduga*. B. Electrophoregram of serum Tranferrin (Trf). Lanes 1, 2 *M. booduga *(M); Lanes 3, 4 *M. booduga *(V); Lanes 5, 6 *M. terricolor *I; Lanes 7, 8 *M*. *terricolor *II; Lanes 9, 10 *M*. *terricolor *III. Lanes 1 to 4 (arrow) show faster allele in *M. booduga*. Samples in lanes 2, 3 are heterozygotes. C. Electrophoregram of Hemoglobin beta chain (Hbb). Lanes 1, 2 *M. booduga *(M); Lanes 3, 4 *M. booduga *(V); Lanes 5, 6 *M. terricolor *I; Lanes 7, 8 *M. terricolor *II; Lanes 9, 10 *M. terricolor *III. Samples in lanes 1 to 4 show major band of faster mobility (arrow) in *M. booduga*.

The sample size studied for the three loci Sod-1, Trf and Hbb varied from 11 to 28. The allelic frequencies were 1.000 for the allele Sod-1^a ^in all the three chromosomal species *M. terricolor *I, II and III (table 2, fig. 2A lanes 5 to 10) and 1.000 for the alternative allele Sod-1^b^, which was markedly anodal, in *M. booduga *(table 2, fig. 2A lanes 1 to 4). In *M. booduga *the frequency of Trf^a^, which was much more anodal to Trf^c ^and Trf^d ^was 0.946 (table 2, fig. 2B lanes 1 to 4) and of Trf^b ^it was 0.054. The frequencies were 0.962, 0.913 and 0.904 for Trf^c ^and 0.038, 0.087 and 0.096 for Trf^d ^in *M. terricolor *I, II and III respectively (table 2, fig. 2B lanes 5 to 10). For heamoglobin beta chain (Hbb), in *M. terricolor *I, II and III, only allele Hbb^p ^was present (table 2, fig. 2C lanes 5 to 10), but in *M. booduga *the frequency of Hbb^p ^was very low being 0.034 whereas it was 0.966 for Hbb^b ^(table 2, fig. 2C lanes 1 to 4).

**Table 2:** Allele frequencies at *Hbb, Trf *and *Sod-1 *loci in the Indian pygmy field mice with sample size in parenthesis.

	*M. booduga (M)*	*M. booduga (V)*	*M. terricolor *I	*M. terricolor *II	*M. terricolor *III
*Hbb*^*p*^	0.067	---	1.000	1.000	1.000
*Hbb*^*b*^	0.933	1.000	---	---	---
N	(15)	(19)	(18)	(15)	(19)

*Trf*^*a*^	0.929	0.963	---	---	---
*Trf*^*b*^	0.071	0.037	---	---	---
*Trf*^*c*^	---	---	0.962	0.913	0.904
*Trf*^*d*^	---	---	0.038	0.087	0.096
N	(28)	(27)	(26)	(23)	(26)

*Sod- 1*^*a*^	---	---	1.000	1.000	1.000
*Sod- 1*^*b*^	1.000	1.000	---	---	---
N	(13)	(26)	(12)	(11)	(12)

## Discussion

There are reports on nest construction in various *Mus *species mostly under laboratory conditions but very few in natural environments [reviewed in [8,9]]. Nest ranges from a simple pallet to a spherical enclosed type and the burrow structures vary from a simple system of *M. m. musculus*, *M. m. domesticus *and *M. m. molossinus *with shallow burrows of 18–20 cm to that of *M. m. hortulanus *which has the most complex of all *Mus *species with its nest chamber 30 to 40 cm deep in soil. The burrows of the co-existing *M*. *booduga *and *M. terricolor *are particularly interesting because of their site of construction. The position of the entrance and exit of the burrows of *M*. *booduga* is located at the highest level while their nest is at the lowest level (fig. 1). On the other hand, *M. terricolor *I, II and III have their nest at the highest level and the entrance and exit at the lowest level of the burrow (fig. 1). This difference is due to their location (in the raised bunds in case of *M. terricolor *I, II and III and below the flat field ground in case of *M*. *booduga*).

Low O_2 _(hypoxic) and high CO_2 _(hypercapnic) conditions have been reported in the occupied burrows of rodents, e.g., *Spalax *and *Geomys *[10-12] and also converging points of evidence show adaptive mechanisms of *Spalax *to the extreme conditions of hypoxia in its subterranean environment [13,14]. In the burrows of the Indian pygmy field mice too relatively less O_2 _and more CO_2 _could be present which would vary with depth, since it is known that proportion of O_2_ decreases and CO_2 _increases with increase in depth below ground level [14-18]. Since post-partum mating takes place in these mice, in the breeding season in the nest chambers litters of different age groups are often found in both *M. booduga *and *M*. *terricolor *burrows. It is quite common that suckling females are already gravid. Increase in the number of individuals in the nest chambers would further affect O_2 _and CO_2 _concentrations. The nest chambers are located at greater depth in *M. booduga *compared to that of *M*. *terricolor *and exchange of air takes place from only one surface (top surface) in the *M*. *booduga *burrows built in flat fields. In the *M*. *terricolor *burrows, on the other hand, exchange of air takes place from three sides of the raised mounds or bunds where they build their burrows. These differences should affect O_2 _and CO_2 _levels and consequently, the burrows and nest chambers of *M*. *terricolor *would be relatively more aerated than that of *M*. *booduga*. Apparently, *M*. *booduga *lives in more hypoxic and hypercapnic conditions compared to *M. terricolor *types I, II and III. F-values obtained by ANOVA also indicate significant difference in the burrow dimensions. Interestingly, very high F-value for depth of the tunnel (table 1) supports significant difference in the level of depth of burrows of *M. budooga *and *M. terricolor *reflecting difference in hypoxic/hypercapnic conditions among burrows.

Hypoxic/hypercapnic conditions known to be lethal to other mammals had no visible effect on these Indian pygmy field mice as also reported in case of subterranean mole rats. However, the evidence shows structural differences in molecules related to hypoxic tolerance like hemoglobin, myoglobin, erythropoietin, VEGF, etc. in subterranean mole rats *Spalax *[14]. Also, evidence from a number of high-altitude vertebrates viz., North American deer mice (*Peromyscus maniculatus*) living in hypoxic conditions indicates that modifications of hemoglobin function typically play a key role in mediating an adaptive response to chronic hypoxia [19,20].

Interestingly, the electrophoretic analysis in the present study showed difference in mobility and fixation of alternative alleles at three loci Hbb, Trf and Sod-1 that are directly or indirectly dependent on oxygen concentration for function, between sibling species *M. booduga *and *M. terricolor*. In *M*. *booduga *the fixed alleles of all the three loci are of faster mobility. An increased anodal mobility of the major component of Hbb has been reported which is associated with increased oxygen affinity [21]. In the serum faster migrating transferrin binds more iron atoms than the slower transferrin, and iron is one of the important components of hemoglobin [22]. Superoxide radical anion is formed even as a normal product of the biological reduction of molecular oxygen. The superoxide dismutase protects organisms against oxygen toxicity by catalyzing dismutation of the reactive radical to hydrogen peroxide and oxygen. Peng et al., [23] have shown in X-irradiated *Drosophila melanogaster *that the allele of slow mobility (S) had higher enzyme activity which provided more protection against radiation damage than allele of fast mobility (F). Their results further indicated a possible adaptive role of the polymorphism (S/F) found in natural populations of *D. melanogaster*. If this could be extrapolated, the allele of faster mobility (Sod-1^b^) present in *M. booduga *may presumably be adapted to an environment with less oxygen in their nests and burrows. Working on the subterranean system *Spalax*, Israeli scientists at University of Haifa are now focusing on whether these rodents could help understand and treat human diseases involving hypoxia [24].

## Conclusion

Thus, location, structure and depth of the burrows might have been the contributory factors for selection of alternative alleles at three loci Sod-1, Trf and Hbb (the molecules related to hypoxic tolerance), which reflect differences in probable adaptive strategy in *M. booduga *and *M. terricolor*. The burrows of the non-overlapping chromosomal species *M. terricolor *I, II and III are however the same which could be due to recency of their evolutionary divergence, but more so because of lack of competition for the well-adapted 'microhabitats' as they are non-overlapping in distribution, unlike between the co-existing, sibling species *M*. *booduga *and *M*. *terricolor *which exhibit mutual "exclusion" of the 'microhabitats' for burrow construction.

Further, understanding the biochemical mechanisms that enable the subterranean rodents to survive and function under conditions of hypoxic stress can provide important insights into the nature of physiological adaptations. The Indian pygmy field mice are a unique model system to study molecular mechanisms for adaptation to hypoxia. The importance of adaptation to hypoxia in understanding the behavior of several biological systems is coming to the fore.

## Methods

### Burrow study

Live burrows in rice and wheat fields were studied by carefully excavating them during breeding seasons which coincided with harvesting of the major crops. Studies of live burrows of *M. booduga *were done from Varanasi in northern India (81 metres above sea level) and Mysore, Erode (770 metres above sea level in between the Eastern and Western ghats, towards south) and Chennai (east side of Eastern ghats 8 metres above sea level) in southern India. Likewise, live burrows of the apparently non-overlapping chromosomal species *M. terricolor *I, II and III were dug up in cultivated fields from Varanasi, Mysore and Chennai. The lengths of the inlet and outlet tunnels, depth of nest chamber and the diameters of nests and inlet/outlet tunnels were measured with precision using measuring tape recorded to the nearest 1 cm.

### Electrophoresis

Tissue sample preparation, buffer systems and stains were similar to those described by Selander et al. [25] and Harris and Hopkinson [26]. Equal amounts of protein (estimated by the method of Lowry et al. [27]) were loaded. Tissue sources were liver for superoxide dismutase (Sod-1), serum for transferrin (Trf) and hemolysate for hemoglobin beta-chain (Hbb). Proteins were resolved on 7.5% polyacrylamide vertical gel. Hemoglobin beta-chain haplotypes were scored according to the method of Morton [28] and transferrin genotypes were scored according to Cohen [29]. Electrophoresis for all the three loci was carried for 4 to 5 hours at a constant 250V at 4°C. For electrophoretic study, *M. booduga *mice were collected from Varanasi and Mysore, and *M. terricolor *I, II and III mice were collected from Varanasi, Mysore and Chennai, respectively. This study has been approved by the institutional ethical committee of Banaras Hindu University.

## Authors' contributions

The study was conceived and planned by TS, the electrophoretic study, data analysis and manuscript preparation was carried out by SS and GN, and NC studied the burrow patterns. All authors have read and approved the manuscript.
